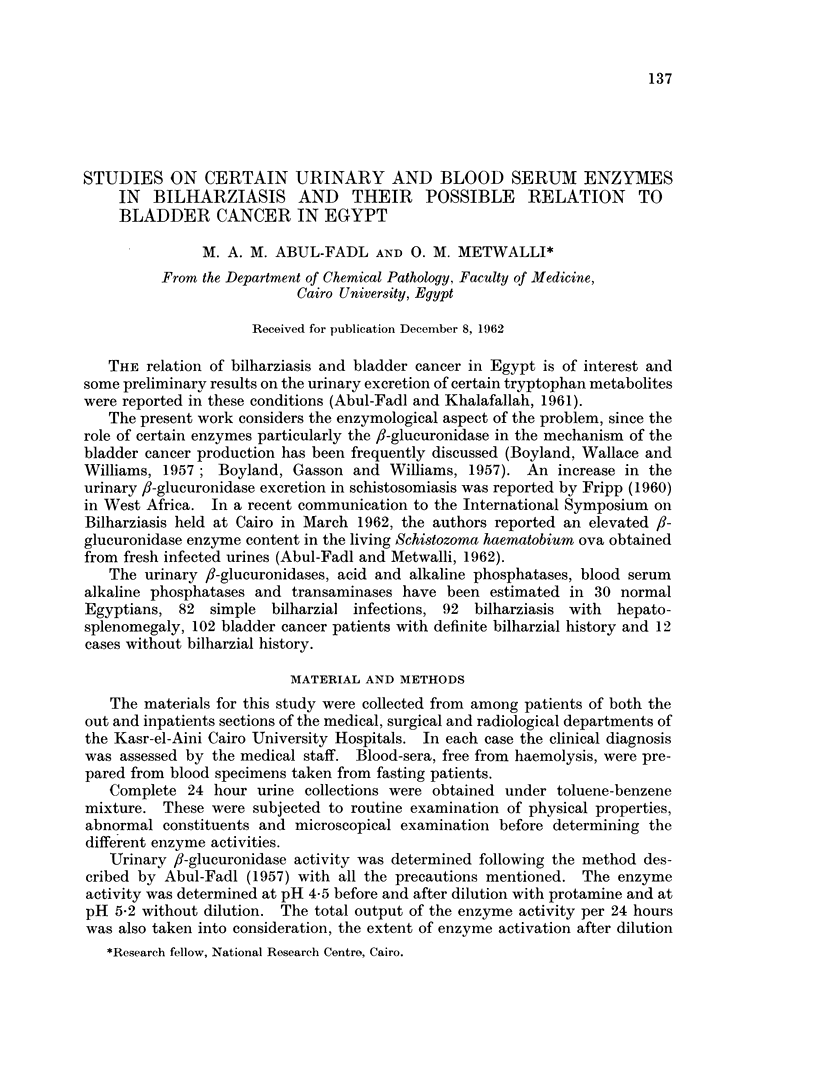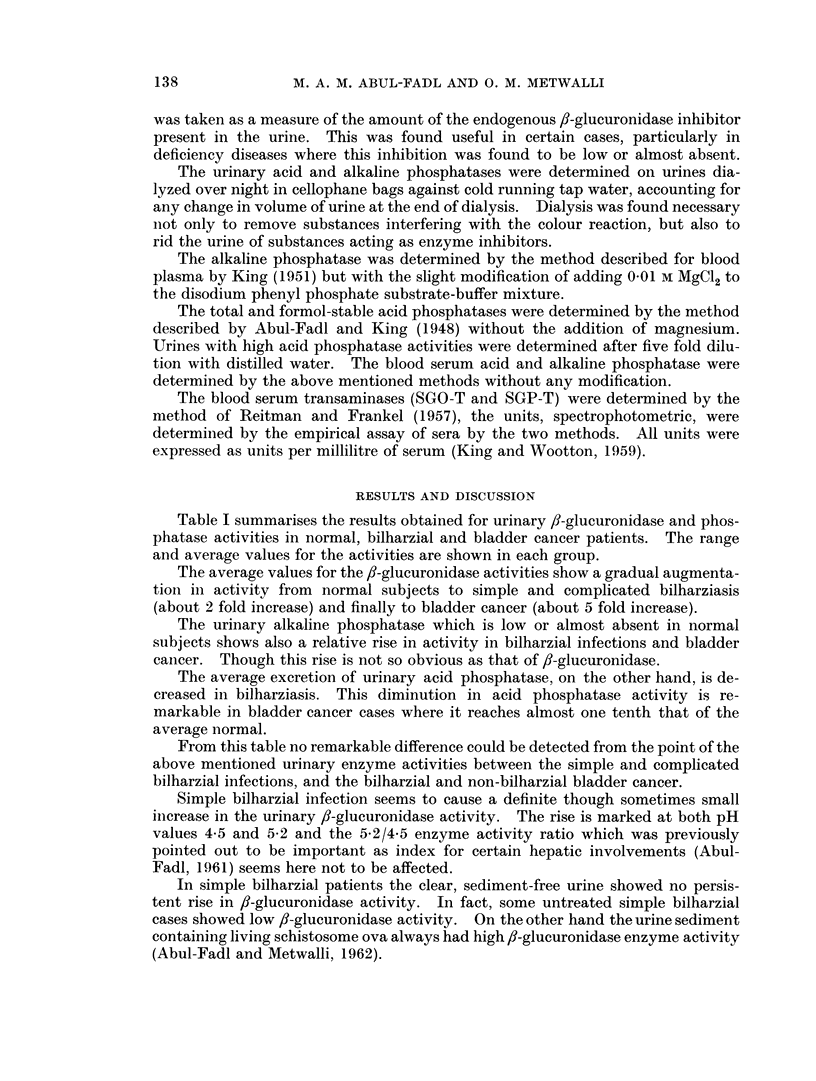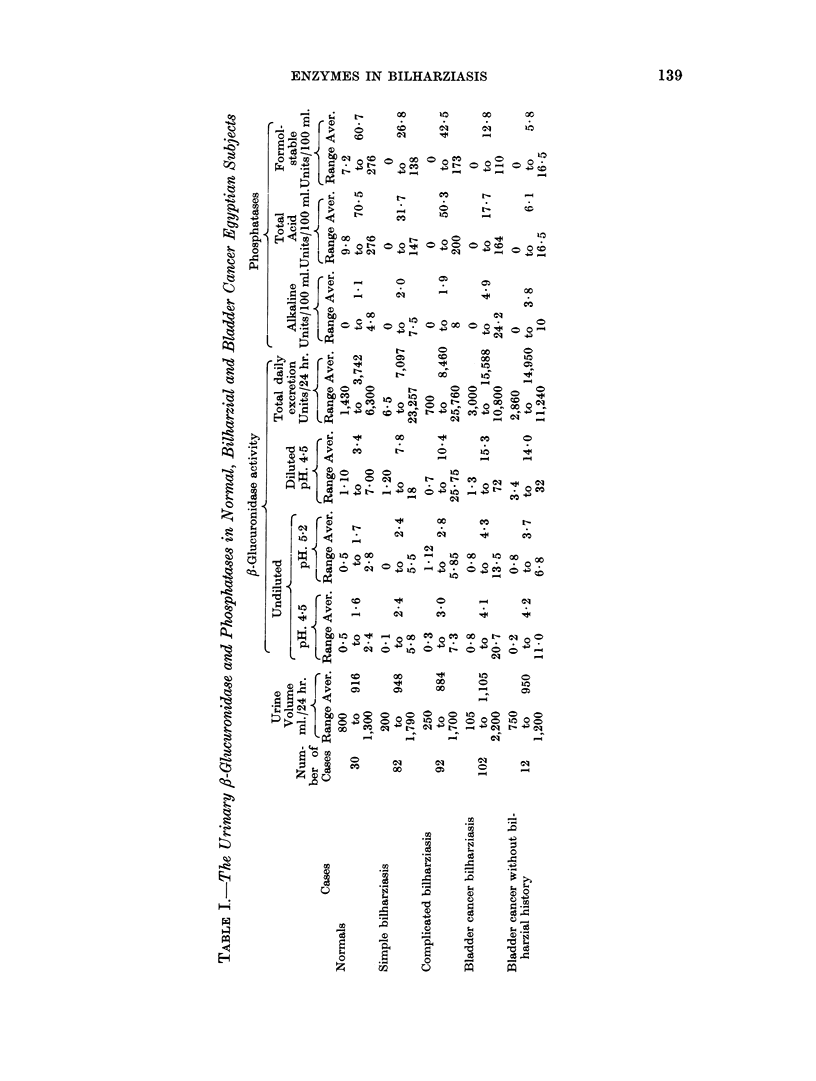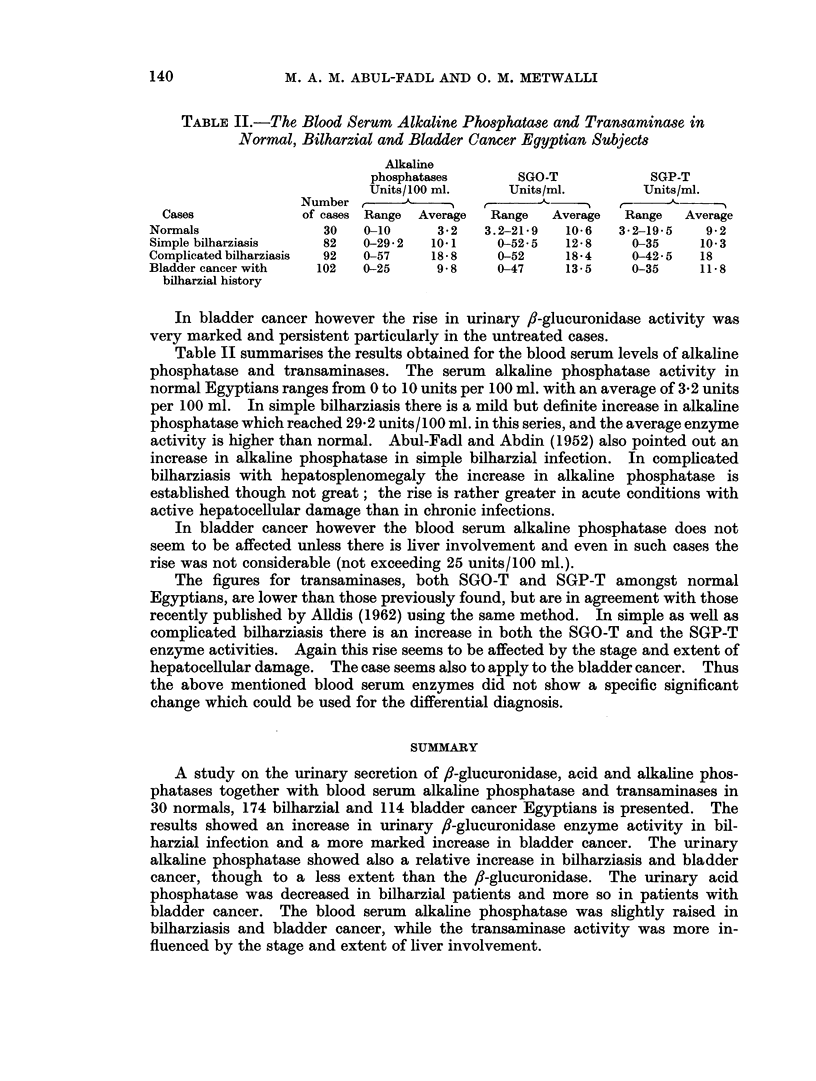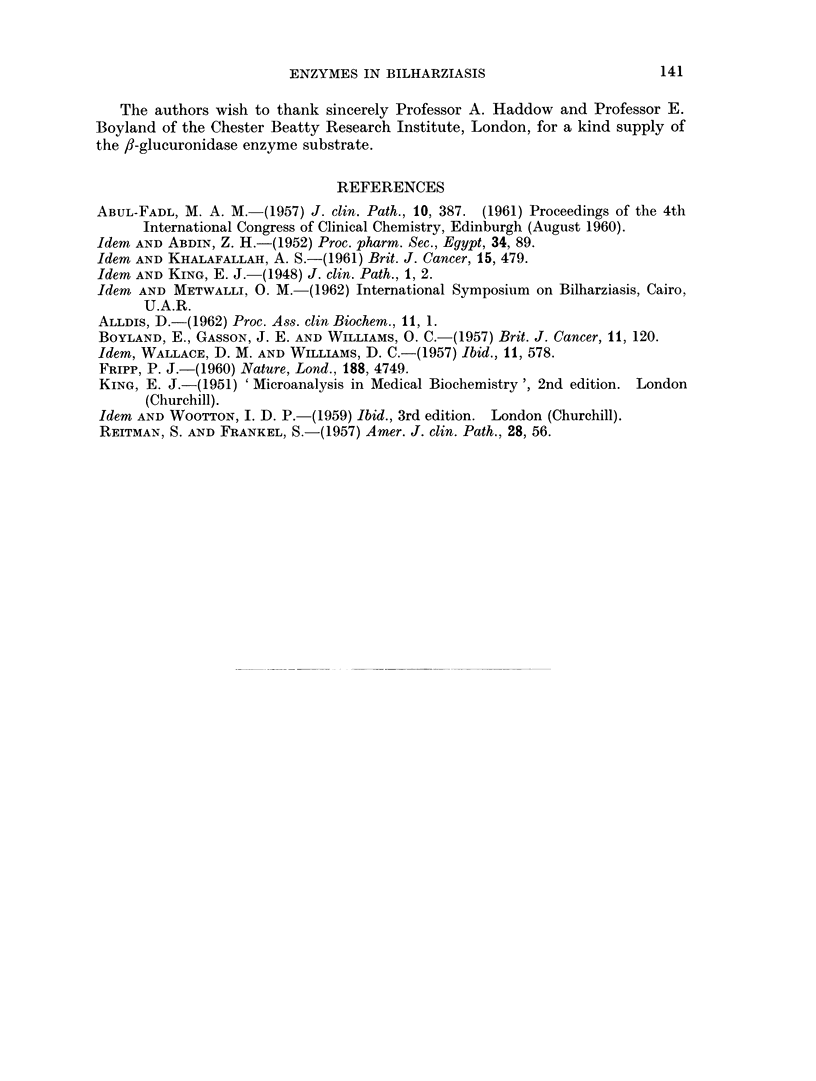# Studies on Certain Urinary and Blood Serum Enzymes in Bilharziasis and their Possible Relation to Bladder Cancer in Egypt

**DOI:** 10.1038/bjc.1963.20

**Published:** 1963-03

**Authors:** M. A. M. Abul-Fadl, O. M. Metwalli


### STUDIES ON CERTAIN URINARY AND BLOOD SERUM ENZYMES IN BILHARZIASIS AND THEIR POSSIBLE RELATION TO BLADDER CANCER IN EGYPT

M. A. M. ABUL-FADL AND O. M. METWALLI*

*From the Department of Chemical Pathology, Faculty of Medicine, Cairo University, Egypt*

Received for publication December 8, 1962

THE relation of bilharziasis and bladder cancer in Egypt is of interest and some preliminary results on the urinary excretion of certain tryptophan metabolites were reported in these conditions (Abul-Fadl and Khalafallah, 1961).

The present work considers the enzymological aspect of the problem, since the role of certain enzymes particularly the $\beta$-glucuronidase in the mechanism of the bladder cancer production has been frequently discussed (Boyland, Wallace and Williams, 1957; Boyland, Gasson and Williams, 1957). An increase in the urinary $\beta$-glucuronidase excretion in schistosomiasis was reported by Fripp (1960) in West Africa. In a recent communication to the International Symposium on Bilharziasis held at Cairo in March 1962, the authors reported an elevated $\beta$-glucuronidase enzyme content in the living *Schistozoma haematobium* ova obtained from fresh infected urines (Abul-Fadl and Metwalli, 1962).

The urinary $\beta$-glucuronidases, acid and alkaline phosphatases, blood serum alkaline phosphatases and transaminases have been estimated in 30 normal Egyptians, 82 simple bilharzial infections, 92 bilharziasis with hepato-splenomegaly, 102 bladder cancer patients with definite bilharzial history and 12 cases without bilharzial history.

### MATERIAL AND METHODS

The materials for this study were collected from among patients of both the out and inpatients sections of the medical, surgical and radiological departments of the Kasr-el-Aini Cairo University Hospitals. In each case the clinical diagnosis was assessed by the medical staff. Blood-sera, free from haemolysis, were prepared from blood specimens taken from fasting patients.

Complete 24 hour urine collections were obtained under toluene-benzene mixture. These were subjected to routine examination of physical properties, abnormal constituents and microscopical examination before determining the different enzyme activities.

Urinary $\beta$-glucuronidase activity was determined following the method described by Abul-Fadl (1957) with all the precautions mentioned. The enzyme activity was determined at pH 4·5 before and after dilution with protamine and at pH 5·2 without dilution. The total output of the enzyme activity per 24 hours was also taken into consideration, the extent of enzyme activation after dilution

*Research fellow, National Research Centre, Cairo.

was taken as a measure of the amount of the endogenous $\beta$-glucuronidase inhibitor present in the urine. This was found useful in certain cases, particularly in deficiency diseases where this inhibition was found to be low or almost absent.

The urinary acid and alkaline phosphatases were determined on urines dialyzed over night in cellophane bags against cold running tap water, accounting for any change in volume of urine at the end of dialysis. Dialysis was found necessary not only to remove substances interfering with the colour reaction, but also to rid the urine of substances acting as enzyme inhibitors.

The alkaline phosphatase was determined by the method described for blood plasma by King (1951) but with the slight modification of adding 0·01 M $MgCl_2$ to the disodium phenyl phosphate substrate-buffer mixture.

The total and formol-stable acid phosphatases were determined by the method described by Abul-Fadl and King (1948) without the addition of magnesium. Urines with high acid phosphatase activities were determined after five fold dilution with distilled water. The blood serum acid and alkaline phosphatase were determined by the above mentioned methods without any modification.

The blood serum transaminases (SGO-T and SGP-T) were determined by the method of Reitman and Frankel (1957), the units, spectrophotometric, were determined by the empirical assay of sera by the two methods. All units were expressed as units per millilitre of serum (King and Wootton, 1959).

### RESULTS AND DISCUSSION

Table I summarises the results obtained for urinary $\beta$-glucuronidase and phosphatase activities in normal, bilharzial and bladder cancer patients. The range and average values for the activities are shown in each group.

The average values for the $\beta$-glucuronidase activities show a gradual augmentation in activity from normal subjects to simple and complicated bilharziasis (about 2 fold increase) and finally to bladder cancer (about 5 fold increase).

The urinary alkaline phosphatase which is low or almost absent in normal subjects shows also a relative rise in activity in bilharzial infections and bladder cancer. Though this rise is not so obvious as that of $\beta$-glucuronidase.

The average excretion of urinary acid phosphatase, on the other hand, is decreased in bilharziasis. This diminution in acid phosphatase activity is remarkable in bladder cancer cases where it reaches almost one tenth that of the average normal.

From this table no remarkable difference could be detected from the point of the above mentioned urinary enzyme activities between the simple and complicated bilharzial infections, and the bilharzial and non-bilharzial bladder cancer.

Simple bilharzial infection seems to cause a definite though sometimes small increase in the urinary $\beta$-glucuronidase activity. The rise is marked at both pH values 4·5 and 5·2 and the 5·2/4·5 enzyme activity ratio which was previously pointed out to be important as index for certain hepatic involvements (Abul-Fadl, 1961) seems here not to be affected.

In simple bilharzial patients the clear, sediment-free urine showed no persistent rise in $\beta$-glucuronidase activity. In fact, some untreated simple bilharzial cases showed low $\beta$-glucuronidase activity. On the other hand the urine sediment containing living schistosome ova always had high $\beta$-glucuronidase enzyme activity (Abul-Fadl and Metwalli, 1962).

TABLE I.—*The Urinary β-Glucuronidase and Phosphatases in Normal, Bilharzial and Bladder Cancer Egyptian Subjects*

| Cases | Number of Cases | Urine Volume ml./24 hr. Range | Urine Volume ml./24 hr. Aver. | β-Glucuronidase activity: Undiluted pH. 4·5 Range | Undiluted pH. 4·5 Aver. | Undiluted pH. 5·2 Range | Undiluted pH. 5·2 Aver. | Diluted pH. 4·5 Range | Diluted pH. 4·5 Aver. | Total daily excretion Units/24 hr. Range | Total daily excretion Units/24 hr. Aver. | Phosphatases: Alkaline Units/100 ml. Range | Alkaline Units/100 ml. Aver. | Total Acid Units/100 ml. Range | Total Acid Units/100 ml. Aver. | Formol-stable Units/100 ml. Range | Formol-stable Units/100 ml. Aver. |
|---|---|---|---|---|---|---|---|---|---|---|---|---|---|---|---|---|---|
| Normals | 30 | 800 to 1,300 | 916 | 0·5 to 2·4 | 1·6 | 0·5 to 2·8 | 1·7 | 1·10 to 7·00 | 3·4 | 1,430 to 6,300 | 3,742 | 0 to 4·8 | 1·1 | 9·8 to 276 | 70·5 | 7·2 to 276 | 60·7 |
| Simple bilharziasis | 82 | 200 to 1,790 | 948 | 0·1 to 5·8 | 2·4 | 0 to 5·5 | 2·4 | 1·20 to 18 | 7·8 | 6·5 to 23,257 | 7,097 | 0 to 7·5 | 2·0 | 0 to 147 | 31·7 | 0 to 138 | 26·8 |
| Complicated bilharziasis | 92 | 250 to 1,700 | 884 | 0·3 to 7·3 | 3·0 | 1·12 to 5·85 | 2·8 | 0·7 to 25·75 | 10·4 | 700 to 25,760 | 8,460 | 0 to 8 | 1·9 | 0 to 200 | 50·3 | 0 to 173 | 42·5 |
| Bladder cancer bilharziasis | 102 | 105 to 2,200 | 1,105 | 0·8 to 20·7 | 4·1 | 0·8 to 13·5 | 4·3 | 1·3 to 72 | 15·3 | 3,000 to 10,800 | 15,588 | 0 to 24·2 | 4·9 | 0 to 164 | 17·7 | 0 to 110 | 12·8 |
| Bladder cancer without bilharzial history | 12 | 750 to 1,200 | 950 | 0·2 to 11·0 | 4·2 | 0·8 to 6·8 | 3·7 | 3·4 to 32 | 14·0 | 2,860 to 11,240 | 14,950 | 0 to 10 | 3·8 | 0 to 16·5 | 6·1 | 0 to 16·5 | 5·8 |

TABLE II.—*The Blood Serum Alkaline Phosphatase and Transaminase in Normal, Bilharzial and Bladder Cancer Egyptian Subjects*

| Cases | Number of cases | Alkaline phosphatases Units/100 ml. | | SGO-T Units/ml. | | SGP-T Units/ml. | |
|---|---|---|---|---|---|---|---|
| | | Range | Average | Range | Average | Range | Average |
| Normals | 30 | 0–10 | 3·2 | 3.2–21·9 | 10·6 | 3·2–19·5 | 9·2 |
| Simple bilharziasis | 82 | 0–29·2 | 10·1 | 0–52·5 | 12·8 | 0–35 | 10·3 |
| Complicated bilharziasis | 92 | 0–57 | 18·8 | 0–52 | 18·4 | 0–42·5 | 18 |
| Bladder cancer with bilharzial history | 102 | 0–25 | 9·8 | 0–47 | 13·5 | 0–35 | 11·8 |

In bladder cancer however the rise in urinary $\beta$-glucuronidase activity was very marked and persistent particularly in the untreated cases.

Table II summarises the results obtained for the blood serum levels of alkaline phosphatase and transaminases. The serum alkaline phosphatase activity in normal Egyptians ranges from 0 to 10 units per 100 ml. with an average of 3·2 units per 100 ml. In simple bilharziasis there is a mild but definite increase in alkaline phosphatase which reached 29·2 units/100 ml. in this series, and the average enzyme activity is higher than normal. Abul-Fadl and Abdin (1952) also pointed out an increase in alkaline phosphatase in simple bilharzial infection. In complicated bilharziasis with hepatosplenomegaly the increase in alkaline phosphatase is established though not great ; the rise is rather greater in acute conditions with active hepatocellular damage than in chronic infections.

In bladder cancer however the blood serum alkaline phosphatase does not seem to be affected unless there is liver involvement and even in such cases the rise was not considerable (not exceeding 25 units/100 ml.).

The figures for transaminases, both SGO-T and SGP-T amongst normal Egyptians, are lower than those previously found, but are in agreement with those recently published by Alldis (1962) using the same method. In simple as well as complicated bilharziasis there is an increase in both the SGO-T and the SGP-T enzyme activities. Again this rise seems to be affected by the stage and extent of hepatocellular damage. The case seems also to apply to the bladder cancer. Thus the above mentioned blood serum enzymes did not show a specific significant change which could be used for the differential diagnosis.

### SUMMARY

A study on the urinary secretion of $\beta$-glucuronidase, acid and alkaline phosphatases together with blood serum alkaline phosphatase and transaminases in 30 normals, 174 bilharzial and 114 bladder cancer Egyptians is presented. The results showed an increase in urinary $\beta$-glucuronidase enzyme activity in bilharzial infection and a more marked increase in bladder cancer. The urinary alkaline phosphatase showed also a relative increase in bilharziasis and bladder cancer, though to a less extent than the $\beta$-glucuronidase. The urinary acid phosphatase was decreased in bilharzial patients and more so in patients with bladder cancer. The blood serum alkaline phosphatase was slightly raised in bilharziasis and bladder cancer, while the transaminase activity was more influenced by the stage and extent of liver involvement.

The authors wish to thank sincerely Professor A. Haddow and Professor E. Boyland of the Chester Beatty Research Institute, London, for a kind supply of the $\beta$-glucuronidase enzyme substrate.

### REFERENCES

Abul-Fadl, M. A. M.—(1957) *J. clin. Path.*, **10**, 387. (1961) Proceedings of the 4th International Congress of Clinical Chemistry, Edinburgh (August 1960).

*Idem* and Abdin, Z. H.—(1952) *Proc. pharm. Sec., Egypt*, **34**, 89.

*Idem* and Khalafallah, A. S.—(1961) *Brit. J. Cancer*, **15**, 479.

*Idem* and King, E. J.—(1948) *J. clin. Path.*, **1**, 2.

*Idem* and Metwalli, O. M.—(1962) International Symposium on Bilharziasis, Cairo, U.A.R.

Alldis, D.—(1962) *Proc. Ass. clin Biochem.*, **11**, 1.

Boyland, E., Gasson, J. E. and Williams, O. C.—(1957) *Brit. J. Cancer*, **11**, 120.

*Idem*, Wallace, D. M. and Williams, D. C.—(1957) *Ibid.*, **11**, 578.

Fripp, P. J.—(1960) *Nature, Lond.*, **188**, 4749.

King, E. J.—(1951) 'Microanalysis in Medical Biochemistry', 2nd edition. London (Churchill).

*Idem* and Wootton, I. D. P.—(1959) *Ibid.*, 3rd edition. London (Churchill).

Reitman, S. and Frankel, S.—(1957) *Amer. J. clin. Path.*, **28**, 56.